# Assessment of the need for routine intraoperative cell salvage in liver transplantation

**DOI:** 10.1590/acb386423

**Published:** 2023-12-01

**Authors:** Claudianne Maia de Farias Lima, Tatyane Oliveira Rebouças, Luciana Maria de Barros Carlos, Juliana Bezerra Frota Oliveira, Eulene Lima da Silva, Janaína Soares Alves, Clébia Azevedo de Lima, Felipe Pantoja Mesquita, Jannison Karly Cavalcante Ribeiro, Pedro Everson Alexandre de Aquino, Denise Menezes Brunetta, José Huygens Parente Garcia, Antonio Brazil Viana

**Affiliations:** 1 Centro de Hematologia e Hemoterapia do Ceará – Fortaleza (CE), Brazil.; 2 Universidade Federal do Ceará – Hospital Universitário Walter Cantídio – Fortaleza (CE), Brazil.

**Keywords:** Liver Transplantation, Blood Transfusion, Autologous, Hemorrhage

## Abstract

**Purpose:**

This study aimed to assess the necessity of routine intraoperative cell salvage in liver transplantations.

**Methods:**

A total of 327 liver transplants performed between 2014 and 2016 was included in the analysis. Patient data, including pre-transplant examinations, intraoperative red blood cell transfusions, and procedural information, were collected.

**Results:**

The median age of the patients was 54 years old, with 67% (219) being male. The most prevalent ABO blood type was O, accounting for 48% (155) of cases. The leading causes of liver disease were hepatitis C (113 cases, 34.6%) and alcohol-related liver disease (97 cases, 29.7%). Out of the 327 liver transplants, allogeneic red blood cell transfusions were administered in 110 cases (34%) with a median of two units of red blood cells per case. Cell salvage was employed in 237 transplants (73%), and successful blood recovery was achieved in 221 cases (93%). Among the group that recovered more than 200 mL of blood, the median volume of recovered blood was 417 mL, with no transfusion of allogeneic blood required. A total of 90 transplants was performed without utilizing cell salvage, and, among these cases, 19 required blood transfusions, with a median of zero units transfused.

**Conclusions:**

This study suggests that routine cell salvage is unnecessary for all liver transplantations. The most suitable indication for its use is in patients presenting with portal vein thrombosis and abnormal creatinine levels.

## Introduction

Liver cirrhosis is considered the final stage of a series of pathological processes in the liver from different causes, such as chronic and autoimmune hepatitis, alcoholism, in addition to metabolic, vascular, and biliary disorders^1^. According to the World Health Organization (WHO), liver cirrhosis is the 18^th^ cause of death globally. In the United States, its prevalence is 250 patients per 100,000 inhabitants^2^, and, in Europe, cirrhosis is responsible for 1.8% of all deaths, which is equivalent to 170,000/year^3^.

The first liver transplant performed in the world was in March 1963, in a 3-year-old child with biliary atresia, who died intraoperatively due to severe hemorrhage^4^. In Brazil, the first transplant was performed in 1968 at the Hospital das Clínicas de São Paulo, at the Medical School of the Universidade de São Paulo. The patient survived for seven days and died due to acute graft rejection and infection^5^.

The last two decades have been marked by a considerable increase in transplant teams in the country. Brazil has become the largest public transplant system globally and the third largest in the number of liver transplants. The Brazilian Unified Health System (Sistema Único de Saúde–SUS) is responsible for more than 95% of all transplants performed in the country, ensuring universal access to health services^6^.

The history of liver transplantation in Ceará, Brazil, began in May 2002, and, currently, more than 1,800 transplants have been performed. These numbers have shown a growing increase in this type of surgery in the state. The Hospital Universitário Walter Cantídio (HUWC) is considered a national and international reference hospital^7^.

One of the forms of autotransfusion is cell salvage (CS). With this technique, the blood lost in the surgical cavity is aspirated, filtered, centrifuged, washed, and the recovered red blood cells are reinfused^8^. The advantages of using this technique are immediate availability, cost reduction, reduced allogeneic transfusion, and reduced risk of disease transmission^9^.

CS is offered to all hospitals of the state by the Hematology and Hemotherapy Center of Ceará (HEMOCE) since 2001, a hemotherapy reference well-known nationally. The surgeries in which CS is used include cardiac, orthopedic, and vascular surgeries, solid organ transplants, and during the care of Jehovah’s witness patients refusing blood transfusion. The role of CS is extremely important, as it minimizes transfusion risks for patients by reducing the number of allogeneic transfusions. HEMOCE provides equipment, material, and a team of on-call nurses to meet demands 24 hours a day.

Some methods used in liver transplantation and the experience of the multidisciplinary team offer the advantage of reducing blood loss and a transfusion rate of less than 30%, contributing to the prevention of hemodynamic instability^10^.

The management of liver transplantation-associated bleeding remains a very important area of study. However, an effort is needed to identify patients at higher risk of bleeding and create strategies to minimize allogeneic transfusion, thus contributing to the survival of patients and reducing hospital stay. The purpose of this study was to evaluate the need for routine intraoperative CS in liver transplantations.

## Methods

A retrospective, descriptive, and quantitative study was conducted using the medical records of patients who underwent liver transplantation at the HUWC from 2014 to 2016. The study aimed to investigate the need for routine intraoperative CS in liver transplantations, with a particular focus on the reduction of perioperative hemorrhage. A total of 327 liver transplantations was included in the analysis, with three cases involving retransplantation. Data collection and management were performed using the electronic data collection and management tool REDCap, hosted at the Universidade Federal do Ceará Hospital Complex. The study protocol received ethical approval from the Ethics Committee of the hospital (certificate no. 64529317.7.0000.5045).

To assess the presence of perioperative bleeding, a bleeding pattern was defined as the recovery of equal to or greater than 200 mL of blood during the transplantation procedure, which corresponds to the volume of a pack of red blood cells. Patient characteristics, pre-transplant examinations, red blood cell transfusions during transplantation, and procedural data were collected and analyzed.

Descriptive statistics, including median, minimum, and maximum values, were calculated for numerical variables such as blood volume and patient age. Categorical data, such as gender and blood type, were presented as frequencies and percentages. The Mann-Whitney’s U test was employed to examine associations between risk factors and intraoperative bleeding, taking into consideration the non-normal distribution of the data. Associations between categorical variables were assessed using Pearson’s χ^2^ test and Fisher’s exact test.

The statistical analysis was performed using GraphPad Prism version 5.0, a widely used software package for statistical analysis and graphing in biomedical research. A significance level of 5% was considered statistically significant. Multivariate analysis was conducted including variables with p-values less than 0.20 to explore further associations.

## Results

### Characterization of the patients

**Figure 1:**
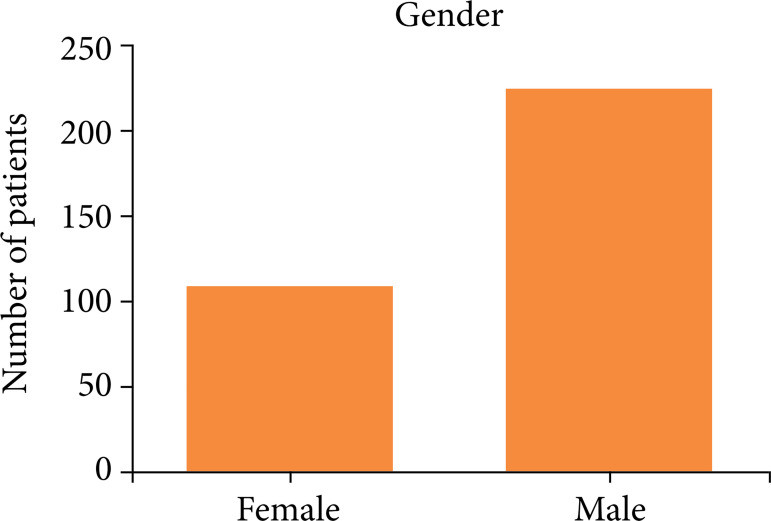
Sample distribution by gender (median age: 54 years old, minimum age: 20, maximum age: 73).

Source: elaborated by the authors.

**Figure 2:**
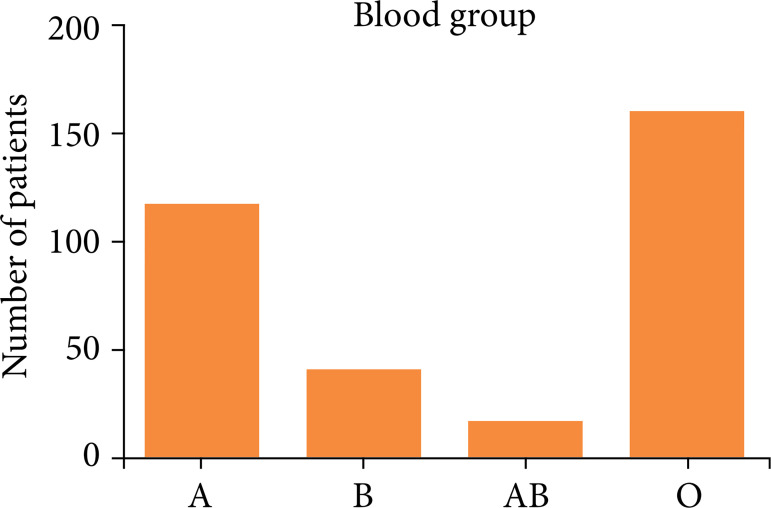
Sample distribution by blood type.

Source: elaborated by the authors.

Among the 327 transplant patients, 222 (67%) were male, and 105 (33%) were female (Fig. 1). The median age was 54 years old, the minimum age was 20, and the maximum was 73. The most prevalent ABO blood type was O, found in 155 (48%) patients, as shown in Fig. 2.

Among the 324 transplant patients, the most prevalent etiology was hepatitis C, accounting for 113 cases (34.6%). Following closely was alcohol-related cirrhosis, observed in 97 cases (29.7%). Additionally, cryptogenic cirrhosis accounted for 43 cases (13.1%), while hepatitis B was identified in 32 cases (9.8%). The remaining etiologies, categorized as “Other etiologies,” were present in 42 cases (12.8%) (Table 1).

**Table 1:** Most frequent etiologies of liver transplants from 2014 to 2016.

Etiologies	n (%)
Hepatitis C	113 (34.6)
Alcoholic cirrhosis	97 (29.7)
Cryptogenic cirrhosis	43 (13.1)
Hepatitis B	32 (9.8)
Other etiologies	42 (12.8)
Total	327 (100)

Source: elaborated by the authors.

### Pre-transplant tests evaluation

Pre-transplant tests were conducted to assess various variables, including hemoglobin levels, platelet count, model for end-stage liver disease (MELD), creatinine levels, international normalized ratio (INR), cold ischemia time (CIT), warm ischemia time (WIT), albumin levels, and volume of recovered blood (Table 2).

**Table 2:** Analysis of pre-transplant tests of all transplant patients.

Variables	Median (min and max)
Hemoglobin	11.5 g/dL (5.8 and 17.1 g/dL)
Platelets	75,850 mm^3^ (16.800 and 529.000 mm^3^)
Model for end-stage liver disease	20 (6 and 40)
Creatinine	0.9 mg/dL (0.3 and 8 mg/dL)
International normalized ratio	1.41 (0.64 and 7.2)
Cold ischemia time	294 min (109 and 628 min)
Warm ischemia time	30 min (18 and 65 min)
Albumin	3.2 g/dL (1.4 and 6.6 g/dL)
Volume of recovered blood	329 (78 and 3.938 mL)

Source: elaborated by the authors.

The results indicate that patients exhibited changes in previous test results, such as anemia, thrombocytopenia, and prolonged INR levels.

### Evaluation of the use of red blood cell transfusion and of the volume of recovered blood from cell salvage

Of the 327 transplants, red blood cell transfusion was performed in 110 (34%) cases, with a median of two packs per transfusion (min: 1; and max: 15). Out the 110 transfused patients, 91 (83%) were treated with CS, and the median volume of recovered blood obtained in 85 (93%) transplants was 389 mL. Among the 327 transplants performed, CS was used in 237 (73%), and the median volume of recovered blood obtained in 221 (93%) cases was 329 mL. However, to assess significant bleeding, we considered those surgeries with a volume of recovered blood higher or equal to 200 mL (Table 3).

**Table 3:** Cell salvage usage by volume of recovered blood vs. no cell salvage usage.

Variables	Volume ≥ 200 mL (%)	Volume < 200 mL (%)	Cell salvage without blood recovery (%)*
Number of transplants	168 (70.9)	53 (22.4)	16 (6.7)
Red blood transfusion	73 (66.3) Median: 0 (min: 0/max: 15)	12 (10.9) Median: 0 (min: 0/max: 7)	6 (5.5) Median: 0 (min: 0/max: 5)
Volume of recovered blood	417 mL (min: 200/max: 3,038)	129 mL (min: 78/max: 199)	

* The cell salvage procedure was performed, but there was no blood recovery. Source: elaborated by the authors.

In 90 transplants, CS was not used, and there was transfusion in 19 (17.3%) cases, with a median of zero (min = 0/max = 6).

### Variables that influenced the volume of recovered blood ≥ 200 mL

Univariate analysis was performed to identify which factors may be related to the greater potential for bleeding, as shown in Table 4.

**Table 4:** Univariate analysis of factors that could influence significant bleeding* #.

Variables	Cell salvage		P-value
	≥ 200 mL (%)	< 200 mL	
Model for end-stage liver disease	22 (20–27)	20 (14–24)	0.012^b^
Port vein thrombosis	18 (10.6)	0 (0)	0.004^d^
Creatinine	1 (0.7–1.6)	0.8 (0.6–1.1)	0.001^b^
Previous surgery	60 (35.3)	17 (33.3)	0.392^c^
Use of anticoagulant	2 (1.2)	0 (0)	> 0.999^d^
International normalized ratio	1.6 (1.3–1.9)	1.4 (1.2–1.7)	0.017^b^
Hemoglobin	10.6 (8.2–12.6)	11.9 (10.5–13.1)	0.006
Platelets	70.2 (46.7–109.7)	76.5 (55.6–119)	0.265
Cold ischemia time	300 (257–384)	276 (239–380.0)	0.001
Warm ischemia time	30 (27–34)	30 (27–35)	0.144

* Data expressed in n (%) and median (25th percentile–75th percentile);

b Mann-Whitney’s test;

c Pearson’s χ^2^ test;

d Fisher’s exact test;

# significance level: p < 0.05.

Source: elaborated by the authors.

The MELD score, presence of portal vein thrombosis, creatinine level, INR, hemoglobin, and cold ischemia time were identified as significant risk factors for bleeding (Table 5). However, in the multivariate analysis, the variable “presence of portal vein thrombosis” was not included due to the phenomenon of quasi-complete separation, as all patients with portal vein thrombosis experienced bleeding above 200 mL. Therefore, it was not possible to estimate parameters for this variable. It is worth noting that the creatinine level emerged as the only significant factor. Additionally, the presence of portal vein thrombosis is an important determinant of increased bleeding.

**Table 5:** Multivariate analysis of factors that could influence significant bleeding* #.

Variable	Odds ratio	95% confidence interval	p-value
Model for end-stage liver disease	1.005	0.9457–1.07	0.868
International normalized ratio	1.266	0.6093–2.63	0.527
Hemoglobin	0.912	0.7770–1.07	0.261
Cold ischemia time	1.003	0.9988–1.01	0.178
Creatinine	1.946	1.016 - 3.725	0.045

* Logistic regression analysis and 95% confidence intervals, outcomes, and predictors;

# significance level: p < 0.05. Source: elaborated by the authors.

## Discussion

In this study, the characteristics of 327 liver transplant patients were analyzed. Most patients were male, and the median age was 54 years old. The leading etiologies for liver transplants were hepatitis C, alcohol-related cirrhosis, cryptogenic cirrhosis, and hepatitis B. Other etiologies also contributed.

Pre-transplant tests revealed notable findings such as anemia, thrombocytopenia, and extended INR. Red blood cell transfusion was performed in a significant number of cases, with CS being used in most of them. The volume of recovered blood during CS was also recorded. Risk factors for bleeding were identified, including the MELD score, presence of portal vein thrombosis, creatinine level, INR, hemoglobin, and cold ischemia time. However, the variable ‘presence of portal vein thrombosis’ could not be included in the multivariate analysis due to limitations. Nevertheless, it remained a critical determinant of increased bleeding.

Similar to our findings, Boin et al.^11^ and Liu et al.^12^ found that hepatitis C virus infection was the main indication for transplantation in their studies. Silva et al.^13^ reported alcoholic cirrhosis as the primary diagnosis in 100 transplanted patients.

While transplantation without blood transfusion is a challenging goal, it has become increasingly possible in recent years. Some studies have reported rates as high as 79.6%^14^. In our study, we observed a 66% rate of transplants without transfusions.

In the study by Massicotte^15^, CS was used in orthotopic liver transplantation patients, allowing for retransfusion in 65% of the cases. Consistent with our findings, the mean volume of retransfused blood was 338 mL. Pre-transplant tests in the aforementioned study were similar to those in our study. In the group with the largest amount of bleeding (≥ 200 mL), the hemoglobin levels were 10.8 vs. 10.6, platelet counts were 95,000 vs. 70,200, INR was 1.8 vs. 1.6, and MELD scores were 17 vs. 22. Notably, both studies obtained a similar MELD value (0.4 vs. 0), despite one group experiencing less severe bleeding than the other. This resulted in a saving of approximately one pack of red blood cells per patient, as the amount of blood recovery in the transplants in which CS was used reached 93%. The median volume of recovered blood was equivalent to approximately two packs of red blood cells^16^.

Studies have cited risk factors for increased bleeding, including the presence of portal vein thrombosis, previous abdominal surgery, inflammatory adhesions after surgery, and prolonged surgical time^17^^–^ 19. However, in our study, these variables did not contribute significantly to increased bleeding, except for the presence of portal vein thrombosis. All patients with portal vein thrombosis had a volume of recovered blood exceeding 200 mL, indicating a higher risk of bleeding^5^.

Cywinski^20^ found a relationship between MELD, bilirubin, creatinine, INR, and platelet count, and an increased risk of bleeding, partially corroborating our study. MELD was a relevant risk factor in the univariate analysis, and creatinine was a risk factor for bleeding in the outcome analysis. However, there was no relationship between platelet count and the risk of bleeding. The same study predicted that each increase of one unit in creatinine results in a 9% increase in the predicted saving of red blood cell concentrate^20^.

It should be noted that the risk of bleeding during transplantation can vary depending on several factors, from the preoperative conditions of the recipient to unforeseen intraoperative events^20^.

While some authors consider that a cell retriever must be used in all transplants since it is impossible to accurately predict which patients will bleed^16^, our findings indicate that it was possible to identify factors that may predict bleeding^5^. However, conflicting results from many studies and the absence of a definitive conclusion highlight the complexity of predicting the need for transfusion in liver transplantation^20^.

CS is currently a common practice during liver transplantation, but it should be used only when considerable blood loss is expected, serving as a complementary method to replace the lost blood proportionally to the volume of bleeding^21^.

Similarly, controlled hypotension is a common practice during liver transplantation, but it should only be used when considerable blood loss is expected, also aiming to replace the lost blood proportionally to the volume of bleeding^22^.

Liver transplantation carries a high risk of bleeding, leading to severe complications and high mortality rates associated with massive transfusion. Therefore, the development of a model to predict transfusion demand is crucial to reduce the use of blood components and improve patient prognosis^23^.

## Conclusions

The utilization of a cell retriever in all liver transplants may not be universally necessary. In our study, we observed that intraoperative blood recovery was successfully achieved in 93% of cases (221 out of 237 transplants). Interestingly, among these cases, only 66.3% of patients required red blood cell transfusion, with a median transfusion requirement of zero. Furthermore, even among the 90 transplants without intraoperative blood recovery, only 17.3% necessitated red blood cell transfusion, again with a median transfusion requirement of zero. These findings suggest that the decision to employ a cell retriever should be carefully considered based on individual patient factors.

Our analysis identified two key variables significantly associated with a higher risk of bleeding during liver transplantation: the creatinine level and the presence of portal vein thrombosis. Although patients with portal vein thrombosis were not included in the multivariate analysis, their condition was noteworthy as all these patients experienced substantial bleeding and had a high volume of recovered blood. These results emphasize the importance of assessing individual patient characteristics when determining the need for intraoperative blood recovery techniques.

Based on our study findings, a more personalized approach to the use of cell retrievers in liver transplantation appears to be warranted. By considering factors such as the creatinine level and the presence of portal vein thrombosis, the application of intraoperative blood recovery techniques can be optimized to minimize unnecessary transfusions and enhance resource utilization.

In conclusion, our study underscores the importance of tailored risk assessment in liver transplantation and challenges the notion of universal cell retriever usage. Further research is necessary to validate these findings and refine the selection criteria for the implementation of intraoperative blood recovery techniques in liver transplantation.

## Data Availability

The data will be available upon request.
